# Supplementary material for: Highly Mutable Linker Regions Regulate HIV-1 Rev Function and Stability
Source: Sci Rep. 2019 Mar 26;9:5139. doi: 10.1038/s41598-019-41582-7 (PMC6435700; doi:10.1038/s41598-019-41582-7)

SUPPLEMENTARY INFORMATION

for

Highly Mutable Linker Regions Regulate HIV-1 Rev Function and Stability

Bhargavi Jayaraman[1,3], Jason D Fernandes[1,#a, 3], Shumin Yang[1,2], Cynthia Smith[1], Alan D Frankel[1,*]

[1]Department of Biochemistry and Biophysics, University of California San Francisco, San Francisco, CA, 94158, USA

[#a]Present Address: UCSC Genomics Institute/Howard Hughes Medical Institute, University of Santa Cruz, Santa Cruz, CA, 95060, USA

[2]School of Medicine, Tsinghua University, Beijing, 100084, China.

[3]These authors contributed equally to this work

*corresponding author: frankel@cgl.ucsf.edu

SUPPLEMENTARY FIGURES CAPTIONS

FIGURE S1: Rev functional assays. A) Reporter Assay: The Gag-Pol proteins are encoded in an intron between a splice donor (SD) and splice acceptor (SA); Rev or a Rev mutant is provided in trans and co-expression of these two constructs results in production of the Gag-Pol product, capsid protein p24 which is measured by ELISA. B) Comparison of Gag-Pol mRNA export, (cytoplasmic Gag-Pol mRNA levels, determined by qPCR and normalized to GAPDH mRNA levels) and p24 protein production from the reporter assay outlined in (A) in the presence of different Rev mutants. mRNA export correlates well with measured p24 protein levels, indicating that p24 expression levels report on the nuclear export activity of Rev, as has been reported previously [1–4]. Data presented are from biological replicates. C) Non-overlapped viral replication assay. A provirus where the endogenous locus of *rev* is ablated and a *rev* mutant is inserted into the *nef* locus is used to produce viral particles in 293T cells (with wild-type *rev* provided in trans to ensure viral particle production). The mutant virus is then used to infect SupT1 cells and viral spread is measured by p24 ELISA. D) Viral replication spread assays comparing NL4-3 *rev-in-nef* virus to the NL4-3 virus.

FIGURE S2: A) Top panel: Reporter activity for a serial 2-fold dilution of transfected Rev plasmid with constant amount of reporter plasmid. Data are mean ± standard deviation (s.d) of biological replicates. Western blots below showing protein expression for Strep-tagged Rev and GAPDH loading control. Bottom panel: Plot of Rev band intensities from the above western blot vs. ng of plasmid transfected, showing that the band intensities are in the linear range for the indicated quantities of Rev. B) Relative Rev mRNA expression levels in 293T cells, determined by qPCR and normalized to GAPDH mRNA levels. Data presented are from biological replicates.

FIGURE S3: Residue 28 in the Turn A) Experimental fitness of residues for position 28 from the CDMS data B) Reporter assay monitoring export activity of mutations at residue 28. Western blots below showing protein expression for Strep-tagged Rev and GAPDH loading control. C) Corresponding viral replication spread experiments. Data are mean ± s.d of biological triplicates. D) Reporter assay monitoring export activity at 25 ng and 2.5 ng of transfected Rev plasmid. Export activity before normalization is shown here while the normalized activity is shown in Figures 6C and 7C.

FIGURE S4: A) Length distributions of common Rev isolates observed in patients and domain organizations of major isolates with coloring as in Figure 1 and numbering according to HXB2 reference sequence. B) Reporter assay monitoring export activity of a ‘QSQGTET’ insertion into

the Rev C-terminus. Western blots below showing protein expression for Strep-tagged Rev and GAPDH loading control.

FIGURE S5: A) Original blot for Figure 3E B) Original blot for Figure 4E C) Original blot for Figure 5G.

References

1 Hadzopoulou-Cladaras M, Felber BK, Cladaras C, Athanassopoulos A, Tse A, Pavlakis GN. The rev (trs/art) protein of human immunodeficiency virus type 1 affects viral mRNA and protein expression via a cis-acting sequence in the env region. *J Virol* 1989; **63**: 1265–74.

2 Emerman M, Vazeux R, Peden K. The rev gene product of the human immunodeficiency virus affects envelope-specific RNA localization. *Cell* 1989; **57**: 1155–65.

3 Felber BK, Hadzopoulou-Cladaras M, Cladaras C, Copeland T, Pavlakis GN. rev protein of human immunodeficiency virus type 1 affects the stability and transport of the viral mRNA. *Proc Natl Acad Sci U S A* 1989; **86**: 1495–9.

4 Hammarskjöld ML, Heimer J, Hammarskjöld B, Sangwan I, Albert L, Rekosh D. Regulation of human immunodeficiency virus env expression by the rev gene product. *J Virol* 1989; **63**: 1959–66.

Figure S1

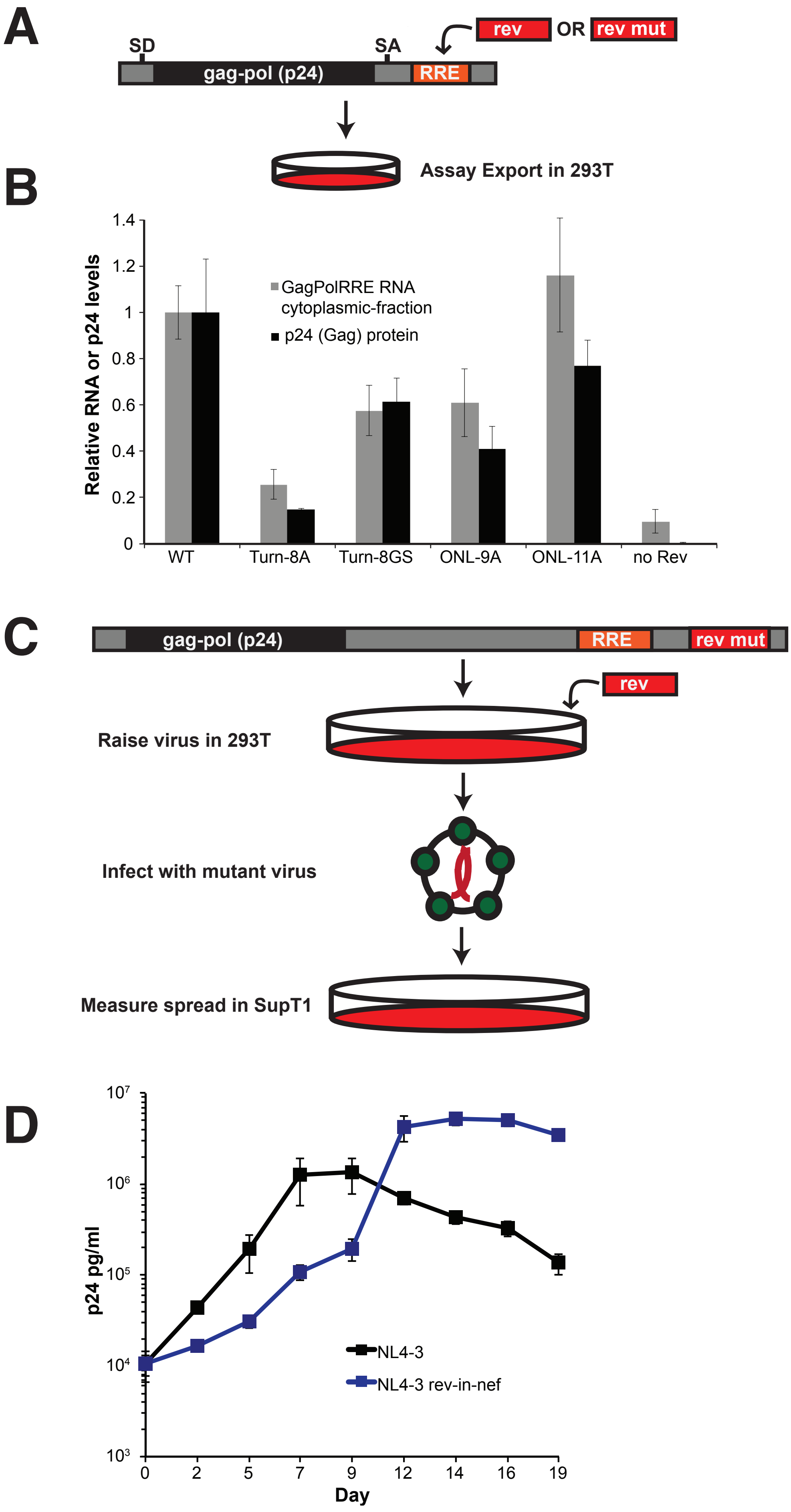
A
SD
SA
gag-pol (p24)
RRE
rev
OR
rev mut
Assay Export in 293T
B
Relative RNA or p24 levels
1.4
1.2
1
0.8
0.6
0.4
0.2
0
GagPolRRE RNA cytoplasmic-fraction
p24 (Gag) protein
WT
Turn-8A
Turn-8GS
ONL-9A
ONL-11A
no Rev
C
gag-pol (p24)
RRE
rev mut
rev
Raise virus in 293T
Infect with mutant virus
Measure spread in SupT1
D
p24 pg/ml
10^7
10^6
10^5
10^4
10^3
NL4-3
NL4-3 rev-in-nef
0
2
5
7
9
12
14
16
19
Day

Figure S2

A

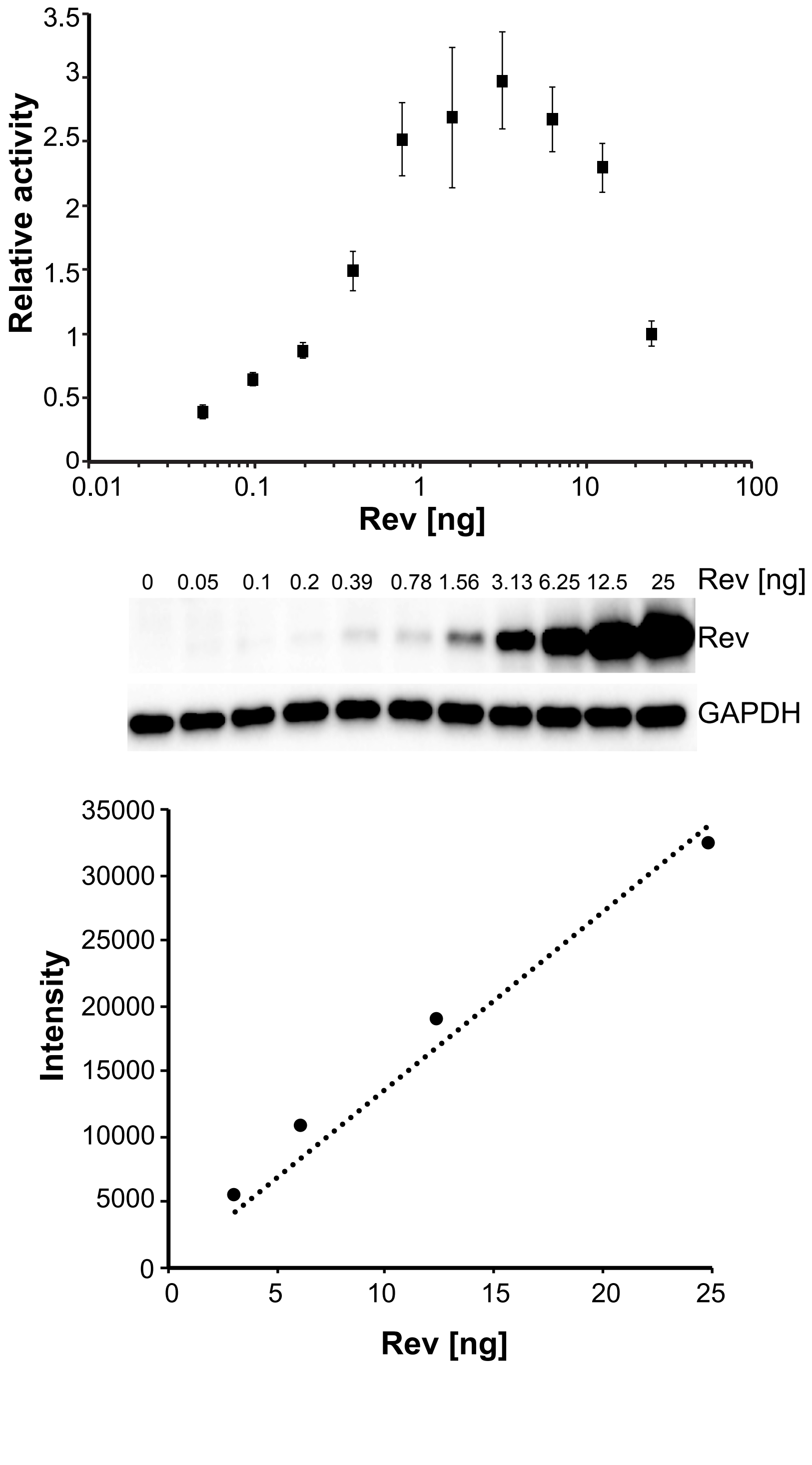

Relative activity
3.5
3
2.5
2
1.5
1
0.5
0
0.01
0.1
1
10
100
Rev [ng]
0
0.05
0.1
0.2
0.39
0.78
1.56
3.13
6.25
12.5
25
Rev [ng]
Rev
GAPDH
Intensity
35000
30000
25000
20000
15000
10000
5000
0
0
5
10
15
20
25
Rev [ng]

B

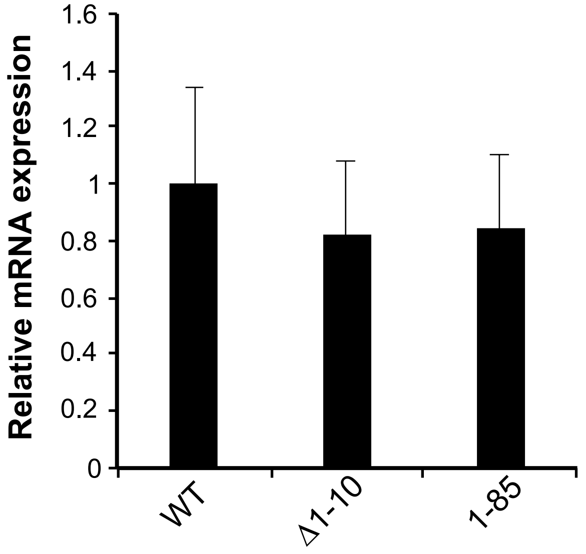

Relative mRNA expression
1.6
1.4
1.2
1
0.8
0.6
0.4
0.2
0
WT
Δ1-10
1-85

Figure S3

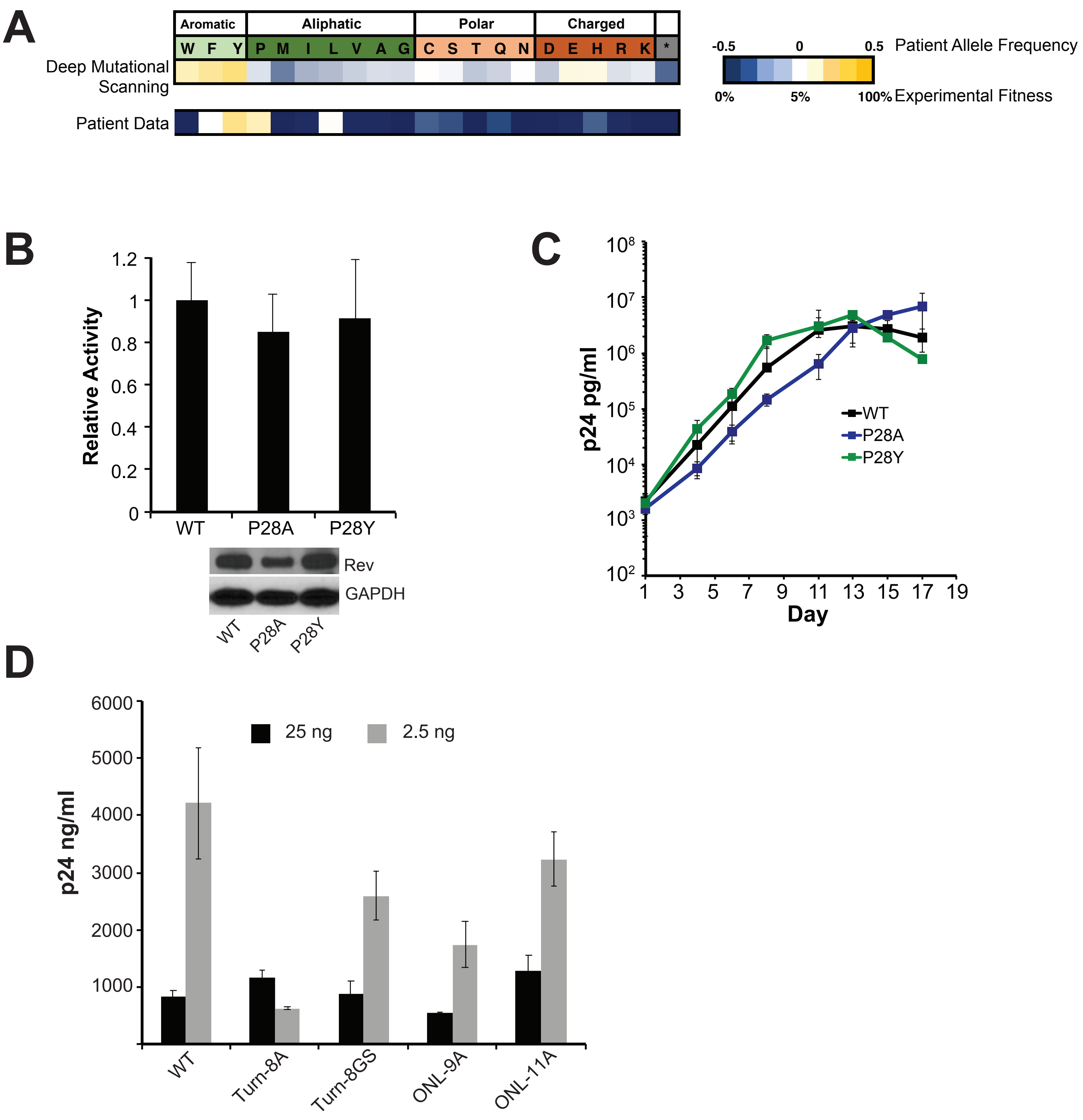
A
Aromatic
Aliphatic
Polar
Charged
W F Y P M I L V A G C S T Q N D E H R K *
Deep Mutational Scanning
Patient Data
-0.5 0 0.5 Patient Allele Frequency
0% 5% 100% Experimental Fitness
B
Relative Activity
1.2
1
0.8
0.6
0.4
0.2
0
WT
P28A
P28Y
Rev
GAPDH
WT
P28A
P28Y
C
p24 pg/ml
10^8
10^7
10^6
10^5
10^4
10^3
10^2
1 3 5 7 9 11 13 15 17 19
Day
WT
P28A
P28Y
D
p24 ng/ml
6000
5000
4000
3000
2000
1000
25 ng
2.5 ng
WT
Turn-8A
Turn-8GS
ONL-9A
ONL-11A

Figure S4

A

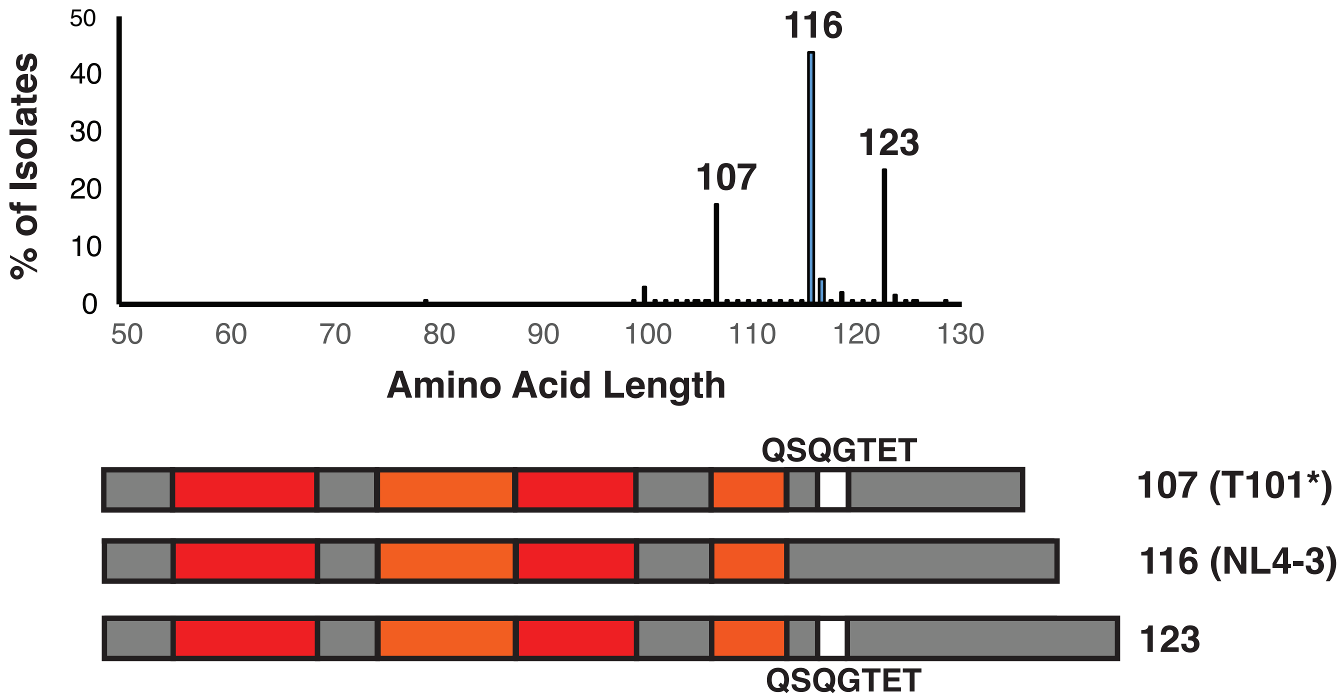
% of Isolates
50
40
30
20
10
0
116
107
123
50
60
70
80
90
100
110
120
130
Amino Acid Length
QSQGTET
107 (T101*)
116 (NL4-3)
123
QSQGTET

B

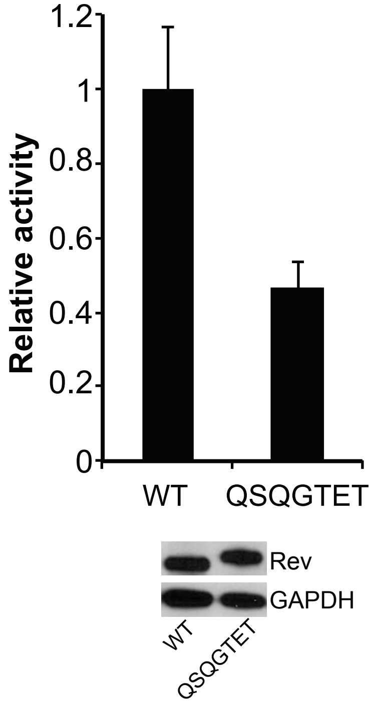
Relative activity
1.2
1
0.8
0.6
0.4
0.2
0
WT
QSQGTET
Rev
GAPDH
WT
QSQGTET

Figure S5

A

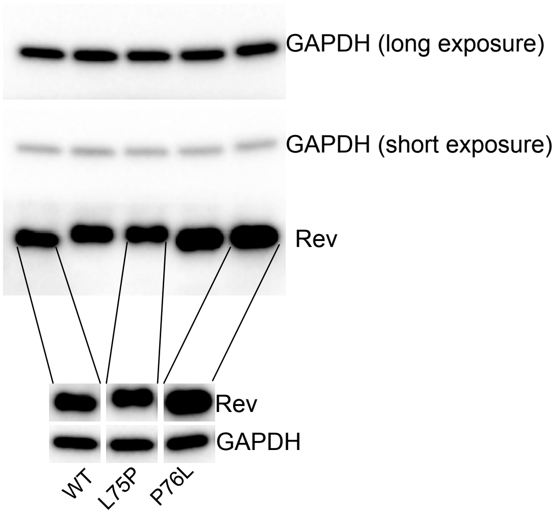

B

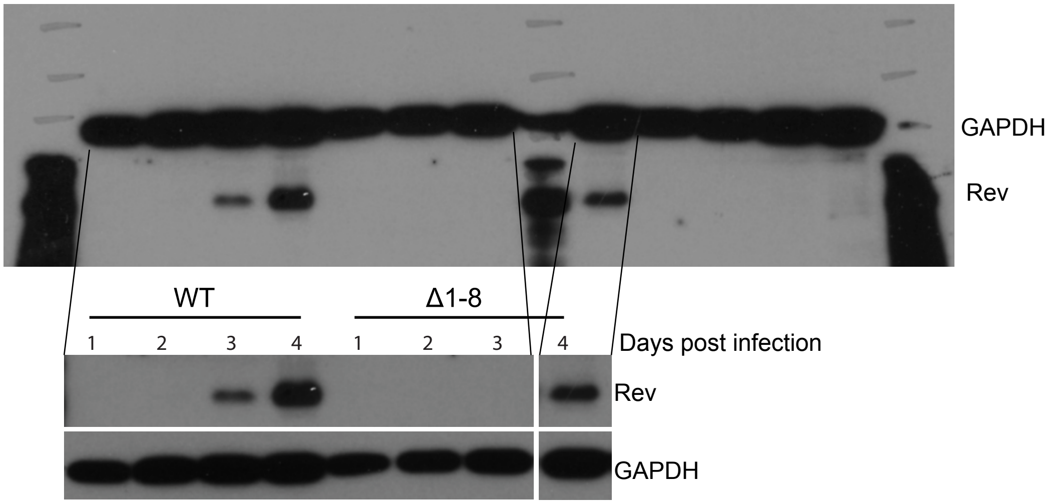

C

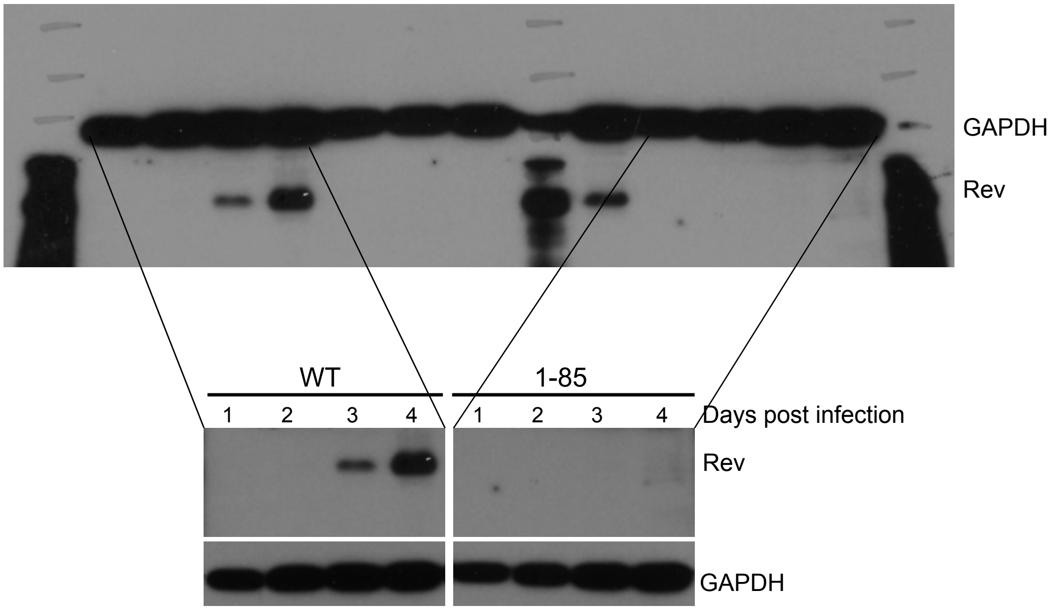

Supplement: Supplementary file 1 — Supplementary information for Highly Mutable Linker Regions Regulate HIV-1 Rev Function and Stability [file 41598_2019_41582_MOESM1_ESM.pdf]
